# Khat chewing and its associated factors among pregnant women in Chiro district, eastern Ethiopia: a community-based study

**DOI:** 10.3389/fpsyt.2023.1253128

**Published:** 2023-11-15

**Authors:** Habtamu Geremew, Fekadu Abera Kebede, Abraham Negash, Misganaw Asmamaw Mengstie, Demeke Geremew

**Affiliations:** ^1^College of Health Science, Oda Bultum University, Chiro, Ethiopia; ^2^Department of Nursing, College of Health Science, Oda Bultum University, Chiro, Ethiopia; ^3^School of Nursing and Midwifery, College of Health and Medical Sciences, Haramaya University, Harar, Ethiopia; ^4^Department of Biochemistry, College of Medicine and Health Sciences, Debre Tabor University, Debre Tabor, Ethiopia; ^5^Immunology and Molecular Biology Unit, Department of Medical Laboratory Sciences, College of Medicine and Health Sciences, Bahir Dar University, Bahir Dar, Ethiopia

**Keywords:** prevalence, khat chewing, addiction, pregnant women, Ethiopia

## Abstract

**Introduction:**

Despite its deleterious consequences, khat chewing is escalating worldwide. However, there is a lack of evidence about the extent of khat chewing among pregnant women in Ethiopia, particularly in the current study area. Therefore, this study aimed to assess the prevalence of current khat chewing and its associated factors among pregnant women in Chiro district, eastern Ethiopia.

**Methods:**

This community-based cross-sectional study was conducted in Chiro district from November 1 to 30, 2022 G.C. Study participants were selected using the systematic random sampling technique. An interview-administered structured questionnaire was used to collect data through a house-to-house survey. The data were entered into EpiData version 3.1 and analyzed in STATA 14 software. Characteristics of study participants were summarized using descriptive analysis, and binary logistic regression was used to identify determinants of khat chewing.

**Results:**

A total of 409 pregnant women participated in this study, with a response rate of 99%. The overall prevalence of khat chewing was 60.4% (95% CI: 55.5%, 65.2%). Religion (AOR: 2.08; 95% CI: 1.13, 3.82), khat cultivation (AOR: 0.43; 95% CI: 0.25, 0.77), partner khat use (AOR: 5.54; 95% CI: 3.11, 9.88), pre-pregnancy khat use (AOR: 9.95; 95% CI: 5.55, 17.81), antenatal care (ANC) visit (AOR: 2.71; 95% CI: 1.41, 5.21), and mental distress (AOR: 4.89; 95% CI: 2.38, 10.02) were significantly associated with current khat chewing.

**Conclusion:**

The majority of pregnant women in the study area practice khat chewing. Thus, accessible and comprehensive pre-conception and pre-natal care incorporating the prevention and management of antenatal khat chewing is crucial to overcome this problem. Provision of mental healthcare involving partners of pregnant women is also important to reduce the extent and impacts of khat chewing during pregnancy. Further longitudinal studies triangulated with qualitative designs are recommended.

## Introduction

Khat (Catha edulis) is an evergreen stimulant plant that is widely cultivated and consumed in East Africa and the Arabian Peninsula (1). The young buds and fresh, tender leaves are chewed to attain psycho-stimulation and euphoria (2). Cathinone and cathine, which are central nervous system stimulants with a qualitatively similar effect as amphetamine, are the principal active ingredients in fresh khat leaves (2–4). In its early stages, khat chewing results in excitement, cheerfulness, relief from fatigue, increased energy, the ability to associate ideas, and high confidence (5, 6). However, these effects are short lived and then replaced by outweighing negative consequences like depression, insomnia, and anxiety (6).

Khat chewing is increasingly becoming a global public health threat (7, 8). Advancing global market and increased production of the crop due to its ability to tolerate climate extremes have enhanced the accessibility and consumption of khat worldwide (8, 9). The practice is widely prevalent in Eastern Africa and the Middle East (1, 8). A cross-sectional study in Yemen found that three in ten adult Yemeni women practice khat chewing (10). Whereas, about 90% of adult men in Yemen exercise khat chewing (11). Another house-to-house survey in Kenya revealed that the prevalence of current khat chewing was 36.8% (12). In Ethiopia, the extent of current khat use ranges from 4 to 64.9%, yet it substantially varies between study populations and geographical areas (13–15).

Khat chewing has multiplex consequences, especially among reproductive-age women (2, 14); these problems are exceedingly escalated, and it is a potential risk for serious reproductive health problems among pregnant women (16). Such problems include preterm labor, pre-labor rupture of membranes, intrauterine fetal death, fetal distress, congenital malformations, anemia, low birth weight, and perinatal death (16–18). It is also associated with preeclampsia, induction of labor, decreased lactation, and embryotoxic effects (19, 20). Thus, indicating its numerous consequences on the health of the mother and her offspring.

Despite its deleterious consequences, khat chewing practice is escalating worldwide (1, 7); its close relation with cultural norms and lack of clear regulatory rules in many high-producing countries have made control efforts less effective, if not ineffective (21, 22). In Ethiopia, measures like increased taxation have been implemented (23), yet the problem remains substantial (24). Although the topic is better investigated among a few segments of the population (24, 25), there is a paucity of evidence about khat chewing practice among pregnant women in Ethiopia, particularly in the current study area. Therefore, this study aimed to assess the prevalence of current khat chewing and its associated factors among pregnant women in Chiro district, eastern Ethiopia.

## Materials and methods

### Study setting

This study was conducted in Chiro district, which is located in West Hararghe Zone, Oromia Regional State, Ethiopia. The district is 326 km away from the national capital, Addis Ababa, in the east direction. It is bordered by Tullo district on the east, Meiso district on the north, Gemechis district on the south, and Guba Koricha on the west. Administratively, Chiro district is divided into 42 kebeles (three urban and thirty-nine rural kebeles). The total estimated population of the district is 314,056 people, of which 10,898 are pregnant women. The district is known for its high production and consumption of khat (9).

### Study design and period

We employed a community-based cross-sectional study from November 1 to 30, 2022 G.C.

#### Population

Source population: all pregnant women in Chiro District.

Study population: all pregnant women in the selected kebeles of Chiro district.

#### Eligibility criteria

All pregnant women who lived in Chiro district for at least 6 months before the survey were eligible for this study. However, pregnant women who were seriously ill and/or had difficulties to communicate were excluded from the study.

#### Sample size and sampling procedure

The sample size was estimated using the single population proportion formula with the assumptions of a 95% confidence interval, 5% margin of error, and Khat chewing prevalence of 37.2% from a previous study in eastern Ethiopia (18). Accordingly, the calculated sample size was 359. After adding a 15% non-response rate, the final sample size was estimated to be 413.

After estimating the sample size, it was allocated proportionally to eight randomly selected kebeles within the district. Then, study participants were selected systematically by using the pregnancy screening registration book of health posts as a sampling frame.

### Data collection and quality control

An interview-administered structured questionnaire that was adapted by reviewing previous literature was utilized to collect the data (5, 14, 26). The questionnaire was first prepared in English and then finalized and administered in Oromiffa. A house-to-house survey was employed to collect the data through face-to-face interview by eight clinical nurses, who are fluent in the local language (Oromiffa), and they were supervised by four B.Sc. nurses.

To ensure the quality of the collected data, 2 days training was given to data collectors and supervisors about the study’s aims and how to approach and interview pregnant women. The questionnaire was also pretested on 30 pregnant women in one of the kebeles of Chiro district, which was not included in the study. In addition, the collected data were verified every day for quality, completeness, and consistency.

### Variables of the study

Dependent variable: our outcome variable of interest was the current khat chewing practice of pregnant women, which was a categorical variable with a yes or no response.

Independent variables: the independent variables include socio-demographic, reproductive, and behavioral characteristics of pregnant women, such as age, residence, religion, educational status, antenatal care (ANC) visit, history of abortion, pre-pregnancy khat use, alcohol use, etc.

### Operational definitions

Current khat chewing: a woman who chewed khat at least once in the last month (14, 27).

Alcohol use: a woman who had ever used alcoholic drinks in her lifetime (27).

Mental distress: the level of mental distress was measured using a contextually validated self-reporting questionnaire (28). A woman was screened as positive for mental distress if she had seven or more yes responses out of the total 20 questions (26, 29).

### Data processing and analysis

After checking for completeness, the data were coded and entered into EpiData version 3.1 software. Then, it was exported to STATA 14 statistical software for further analysis. Descriptive analysis like frequency and percentage was performed and presented in tables. Binary logistic regression was used to identify the determinants of khat chewing. Bi-variable logistic regression with a *P*-value less than 0.25 was used to select candidate variables for multivariable analysis. In the multivariable logistic regression model, the association between khat chewing and predictor variables was summarized using the adjusted odds ratio (AOR) and its 95% confidence intervals (CI). Besides, the fitness of the final model was verified using the Hosmer-Lemeshow goodness-of-fit test and classification table.

## Results

### Scio-demographic characteristics of respondents

A total of 409 pregnant women participated in the study, with a response rate of 99%. The median age of the study participants was 26 years (IQR ± of 9 years). The majority of the respondents were rural dwellers (84.8%), married (96.8%), and Muslim religion followers (69.7%), (Table 1).

**TABLE 1:** Scio-demographic characteristics of pregnant women.

Variables	Category	Frequency	Percentage
Age group	15–24	164	40.1
	25–34	185	45.2
	35–45	60	14.7
Residence	Urban	62	15.2
	Rural	347	84.8
Marital status	Married	396	96.8
	Divorced	10	2.5
	Widowed	3	0.7
Religion	Muslim	285	69.7
	Orthodox	110	26.9
	Others^a^	14	3.4
Ethnicity	Oromo	391	95.6
	Others^b^	18	4.4
Educational status	No formal education	173	42.3
	Primary	204	49.9
	Secondary and above	32	7.8
Occupation	Housewife	271	66.3
	Farmer	108	26.4
	Others^c^	30	7.3
Income category	Highest	84	20.5
	Middle	172	42.1
	Lowest	153	37.4
Time to reach the market	≤ 30 min	158	38.6
	30–60 min	167	40.8
	> 60 min	84	20.6

Key: Others^a^: Catholic, Protestant, or Wake Feta; Others^b^: Amhara, Gurage, or Sidama; Others^c^: daily laborer, government employee, or merchant.

### Reproductive features of pregnant women

Most (73.1%) of pregnant women had at least one ANC follow-up during their current pregnancy. More than half (53.3%) of women were in their second trimester, and about one in ten (12.0%) women had a history of abortion (Table 2).

**TABLE 2:** Reproductive features of pregnant women.

Variable	Categories	Frequency	Percentage
Polygamy	Yes	34	8.3
	No	375	91.7
Family size	≤ 4	206	50.4
	> 4	203	49.6
Is this pregnancy planned	Yes	290	70.9
	No	119	29.1
Gravida	≤ 2	136	33.3
	3–4	142	34.7
	≥ 5	131	32.0
ANC follow-up	Yes	299	73.1
	No	110	26.9
Stage of pregnancy	First trimester	56	13.7
	Second trimester	218	53.3
	Third trimester	135	33.0
History of abortion	Yes	49	12.0
	No	360	88.0

### Behavioral characteristics of pregnant women

About two-thirds of pregnant women had partners who practiced khat chewing. Most of the respondents exercised khat cultivation (58.4%), and had a pre-pregnancy khat chewing history (65.3%), (Table 3).

**TABLE 3:** Behavioral characteristics of pregnant women.

Variable	Categories	Frequency	Percentage
Partner chew khat	Yes	269	65.8
	No	140	34.2
History of Khat chewing	Yes	267	65.3
	No	142	34.7
Ever drink alcohol	Yes	124	30.3
	No	285	69.7
Khat cultivation	Yes	239	58.4
	No	170	41.6
Mental distress	Yes	88	21.5
	No	321	78.5

### Khat chewing and associated factors

**FIGURE 1:**
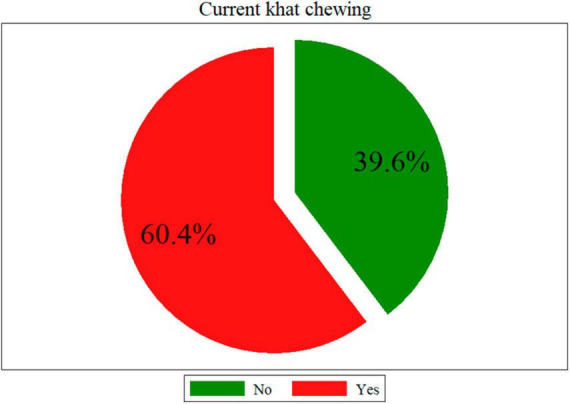
Prevalence of current khat chewing among pregnant women in Chiro district, eastern Ethiopia.

In this study, 60.4% (95% CI: 55.5%, 65.2%) of pregnant women practice khat chewing (Figure 1). The bi-variable analysis indicated that residence, income, ANC visit, ethnicity, religion, educational status, partner khat use, pre-pregnancy khat chewing, khat cultivation, history of abortion, and mental distress had a *P*-value less than 0.25. Whereas, the multivariable logistic regression showed that religion, ANC visit, partner khat use, pre-pregnancy khat chewing, khat cultivation, and mental distress were significantly associated with current khat chewing with a *P*-value less than 0.05.

Correspondingly, women who were Muslim religion followers had 2.08 (95% CI: 1.13, 3.82) times higher odds of khat chewing as compared to Orthodox religion followers. Similarly, khat cultivation increases the likelihood of khat chewing by 57% (AOR: 0.43; 95% CI: 0.25, 0.77). The risk of khat chewing was 5.54 (95% CI: 3.11, 9.88) times higher among women who have khat chewer partners. The likelihood of current khat chewing was 9.95 (95% CI: 5.55, 17.81) times higher among women who were pre-pregnancy khat chewers than their counterparts. Pregnant women who had no ANC visit had a 2.71 (95% CI: 1.41, 5.21) times higher chance of khat chewing than women with at least one ANC visit for their current pregnancy. The odds of khat chewing were 4.89 (95% CI: 2.38, 10.02) times higher among women with mental distress than women without mental distress (Table 4).

**TABLE 4:** Factors associated with khat chewing among pregnant women in Chiro district, eastern Ethiopia.

Variable	Categories	Khat chewing		COR	AOR (95% CI)
		**No**	**Yes**		
Residence	Urban	40 (64.5)	22 (35.5)	0.30	0.51 (0.23, 1.14)
	Rural	122 (35.2)	225 (64.8)	1	1
Religion	Muslim	94 (33.0)	191 (67.0)	2.35	2.08 (1.13, 3.82)*
	Orthodox	59 (53.6)	51 (46.4)	1	1
	Others^a^	9 (64.3)	5 (35.7)	0.64	1.62 (0.32, 8.25)
Khat cultivation	Yes	70 (29.3)	169 (70.7)	1	1
	No	92 (54.1)	78 (45.9)	0.35	0.43 (0.25, 0.77)*
Partner khat use	Yes	66 (24.5)	203 (75.5)	6.71	5.54 (3.11, 9.88)*
	No	96 (68.6)	44 (31.4)	1	1
Pre-pregnancy khat use	Yes	58 (21.7)	209 (78.3)	9.86	9.95 (5.55, 17.81)*
	No	104 (73.2)	38 (26.8)	1	1
ANC follow-up	Yes	139 (46.5)	160 (53.5)	1	1
	No	23 (20.9)	87 (79.1)	3.29	2.71 (1.41, 5.21)*
History of abortion	Yes	14 (28.6)	35 (71.4)	1.75	2.03 (0.81, 5.10)
	No	148 (41.1)	212 (58.9)	1	1
Mental distress	Yes	19 (21.6)	69 (78.4)	2.92	4.89 (2.38, 10.02)*
	No	143 (44.6)	178 (55.4)	1	1

Key: ANC, antenatal care; AOR, adjusted odds ratio; CI, confidence interval; COR, Crud odds ratio, Others^a^: Catholic, Protestant, or Wake Feta; *Significant at *P* < 0.05.

## Discussion

Determining the extent and risk factors of khat chewing among pregnant women is important for public health planning and care. In reference to this, we assessed the prevalence and factors associated with current khat chewing among pregnant women in Chiro district, eastern Ethiopia. Accordingly, 60.4% of pregnant women practice khat chewing. Religion, khat cultivation, having a khat chewer partner, pre-pregnancy khat chewing, ANC visit, and mental distress were significantly associated with current khat chewing.

The overall prevalence of current khat chewing among pregnant women in the present study was in agreement with a previous report from Hossana, southern Ethiopia, 58% (22). However, the magnitude of khat chewing in this study was quite higher than previous reports from eastern Ethiopia, 15.5% (5); and southern Ethiopia, 9.9% (26). This might be due to the high production of khat in the current study area, thereby enhancing its accessibility and consumption (9). On the other hand, the prevalence of khat chewing in our study was lower than previous reports from Yemen, 90% (11); and Kenya, 81% (30). The possible explanation for this variation could be due to the difference in study population: that the estimate in Yemen and Kenya was among the general adult population, including men, and it is evidenced elsewhere that men had a higher risk of khat chewing than women (15).

In line with previous studies (14, 22), our analysis indicated that Muslim religion followers had higher odds of khat chewing. This might be due to the utilization of khat during religious rituals, as evidenced by a previous qualitative exploration (31). The likelihood of khat chewing was also higher among pregnant women who are from households that cultivate khat as compared to their counterparts. The possible explanation could be due to the easy availability and frequent exposure to the crop among women who cultivate it.

The present study found higher odds of khat chewing among pregnant women who had a khat chewer partner than their counterparts. This association was also detected by previous studies (22, 26), and might be due to the need for increasing intimacy and socialization with their partners (1). Similar to previous reports (32), the likelihood of khat chewing was higher among pregnant women with a history of pre-pregnancy khat chewing than among women without such a history. This might be due to the development of psychological dependency and addiction among khat chewers (33). Poor knowledge about the feto-neonatal and maternal consequences of antenatal khat chewing could also be another reason (16).

Our analysis found that attending ANC reduces the likelihood of khat chewing during pregnancy. Accordingly, pregnant women who had no ANC visit for the current pregnancy were more likely to practice khat chewing than women who had at least one ANC visit. This could be partly because women who do not attend ANC may not be informed about the harmful consequences of khat chewing. Hence, indicating the comprehensive role of ANC on maternal and child health. Consistent with previous studies (26, 34), the odds of khat chewing were higher among pregnant women with mental distress as compared to those without it. This might be to use the early euphoric and psycho-stimulant effect of khat as a remedy for the experienced mental distress, yet not understanding or ignoring its enormous negative consequences (16, 35). On the other hand, this might be due to the psychiatric consequences of khat chewing (21). Hence, calling for mental health and psychosocial support for khat chewers.

This study has certain limitations. The cross-sectional nature of the study makes it difficult to establish a causal association. Feto-maternal outcomes of pregnant women were not assessed in relation to their khat chewing pattern. Besides, despite the close tie between khat chewing and cultural norms, our study was not triangulated with a qualitative design.

## Conclusion

The majority of pregnant women in the study area practice khat chewing. Religion, khat cultivation, partner khat use, pre-pregnancy khat use, ANC visit, and mental distress were significantly associated with current khat chewing. Thus, accessible and comprehensive pre-conception and pre-natal care incorporating the prevention and management of antenatal khat chewing is crucial to overcome this problem. Provision of mental healthcare involving partners of pregnant women is also important to reduce the extent and impacts of khat chewing during pregnancy. Further longitudinal studies triangulated with qualitative designs are recommended.

## Data availability statement

The original contributions presented in this study are included in this article/supplementary material, further inquiries can be directed to the corresponding author.

## Ethics statement

Ethical approval was granted by the Oda Bultum University Ethical Review Committee. The study was performed in accordance with the declaration of Helsinki’s ethical principles for medical research involving human subjects. Informed consent was obtained from all pregnant women who participated in this study after a detailed explanation of the purpose of the research and the right to withdraw from the study at any time. Besides, confidentiality of the collected information was assured by not recording women’s personal identifiers.

## Author contributions

HG conceived the study. HG, FK, AN, MM, and DG designed the study, supervised data collection, analyzed the data, wrote the first draft, and critically reviewed the manuscript. All authors have read and approved the final manuscript.
